# Defect induced changes on the excitation transfer dynamics in ZnS/Mn nanowires

**DOI:** 10.1186/1556-276X-6-228

**Published:** 2011-03-16

**Authors:** Uwe Kaiser, Limei Chen, Sebastian Geburt, Carsten Ronning, Wolfram Heimbrodt

**Affiliations:** 1 Department of Physics and Material Sciences Center, Philipps-University of Marburg, Renthof 5, 35032 Marburg, Germany; 2 Institute for Solid State Physics, Friedrich Schiller University of Jena, Max-Wien-Platz 1, 07743 Jena, Germany

## Abstract

Transients of Mn internal 3*d*^5 ^luminescence in ZnS/Mn nanowires are strongly non-exponential. This non-exponential decay arises from an excitation transfer from the Mn ions to so-called killer centers, i.e., non-radiative defects in the nanostructures and is strongly related to the interplay of the characteristic length scales of the sample such as the spatial extensions, the distance between killer centers, and the distance between Mn ions. The transients of the Mn-related luminescence can be quantitatively described on the basis of a modified Förster model accounting for reduced dimensionality. Here, we confirm this modified Förster model by varying the number of killer centers systematically. Additional defects were introduced into the ZnS/Mn nanowire samples by irradiation with neon ions and by varying the Mn implantation or the annealing temperature. The temporal behavior of the internal Mn^2+ ^(3*d*^5^) luminescence is recorded on a time scale covering almost four orders of magnitude. A correlation between defect concentration and decay behavior of the internal Mn^2+ ^(3*d*^5^) luminescence is established and the energy transfer processes in the system of localized Mn ions and the killer centers within ZnS/Mn nanostructures is confirmed. If the excitation transfer between Mn ions and killer centers as well as migration effects between Mn ions are accounted for, and the correct effective dimensionality of the system is used in the model, one is able to describe the decay curves of ZnS/Mn nanostructures in the entire time window.

## Introduction

In semiconductor systems, many processes, such as energy transfer processes, will be modified in nanostructures with respect to bulk semiconductors because of geometric restrictions. The excellent luminescence properties of bulk ZnS/Mn are well-known for more than 50 years and are employed in TFEL displays etc. [1]. The photoluminescence (PL) spectrum of ZnS/Mn alloys is dominated by the internal Mn 3*d*^5 ^luminescence band around 2.15 eV in the yellow range of the visible spectrum. The excitonic emission in the band gap range of ZnS can be entirely quenched on incorporating high amounts of Mn (> 1%) [2] This is due to the efficient energy transfer from the excitonic states into the Mn system. The Mn internal transition takes place between the ^4^T_1 _first excited state of the Mn^2+ ^3*d*^5 ^shell and its ^6^A_1 _ground state. The transient of the luminescence of the Mn internal transition of an isolated Mn ion on a cation site in a defect-free ZnS host matrix is a single exponential decay with a very long intrinsic life time of 1.8 ms [3], which was observed in a very high-quality ZnS/Mn crystal with a Mn concentration in the low doping range, i.e., smaller than 10^17 ^cm^-3 ^and even much lower defect densities. When the ZnS/Mn crystal contains defects or when the Mn concentration is higher, one observes a non-exponential PL decay [4]. The reason is that an energy transfer from the excited Mn ions to defect-related non-radiative sites ("killer center") takes place. This means that in bulk material, the temporal PL behavior is determined by the interplay of two characteristic length scales, i.e., the mean Mn-Mn distance and the mean distance between Mn ions and killer centers. Both length scales are typically in the nanometer range. Thus, artificial structuring on these length scales will impose additional geometrical restrictions that will affect the temporal behavior of the internal Mn^2+ ^(3*d*^5^) PL. In a nanowire, nanoribbon, or nanosphere sample of Mn-doped ZnS nanostructures, the non-exponential decay behavior is influenced by the nanostructure geometry and can therefore be tuned by the sample morphology. We have modified the original Förster model [5] to be applicable for low dimensionality, i.e., 1D and 2D. By considering excitation transfer between Mn ions and killer centers as well as migration effects between Mn ions and using the correct dimensionality, we were able to describe the size and concentration dependence of the decay curves of ZnS/Mn nanostructures, such as wires and ribbons, from a few microseconds to several milliseconds [6]. In this work, we confirm this modified Förster model for different dimensionalities by varying the defect concentration systematically in samples of the same morphology and equal Mn concentrations.

## Sample preparation

**Figure 1:**
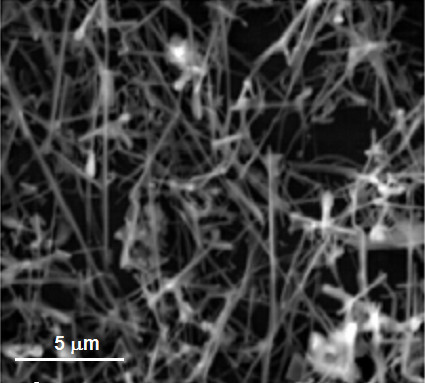
**SEM image of the Mn-implanted ZnS wires**. ZnS wires with diameters of 100-300 nm and lengths of several 10 μm.

ZnS nanowire samples were synthesized by the vapor-liquid-solid method [7]. In the center of a three-zone tube furnace, ZnS powder (99.9% purity) was evaporated, and the vapor was carried by an argon gas flow to silicon (100) substrates coated with 5 nm Au at the lower temperature end of the furnace. The Au film decomposes into liquid Au dots at elevated temperatures which serve as a catalyst for the nanowire growth. The synthesized ZnS wires had a typical length of several micrometers and were about 100 to 300 nm in diameter (see Figure 1). After transferring a thin layer of ZnS nanowires on top of clean Si substrates, the ZnS nanowires were ion-beam implanted with ^55^Mn. A homogeneous Mn distribution over a depth of 300 nm was achieved by using multiple ion energies ranging from 20 to 380 keV and with two different total ion fluences resulting in Mn concentrations of 2.8·10^-3 ^at.% and 0.28 at.%, respectively. There are three different approaches used in order to control the density of killer centers.

1. The number of implantation defects remaining after the Mn implantation can be easily controlled by the implantation temperature. Implantation of Mn ions at higher temperatures enables a higher dynamic annealing of defects during the implantation, as the recombination rate of the ion impact-related vacancy-interstitial pairs is thermally activated [8]. Thus, the number of killer centers remaining after implantation will decrease with increasing implantation temperature (approach 1).

2. Post-annealing of the sample at various temperatures also changes the number of defects in the lattice. Annealing at 600°C in vacuum for 30 min after the Mn implantation process removes most of the remaining defects of the Mn implantation in the ZnS/Mn nanowire system [9], while annealing at lower temperature leaves a higher number of defects in the sample (approach 2).

3. After the Mn implantation process and annealing at 600°C, the ZnS/Mn wire samples were irradiated again, but with neon ions, and without any further annealing. The neon energies were adjusted to create a damage profile matching the Mn implantation depth. Neon as a noble gas atom does not form bonds in the ZnS/Mn lattice, and the majority diffuses out of the sample. Thus, only implantation defects remain in the nanowires, which act as killer centers, and their density can be precisely controlled by the neon implantation fluence (approach 3).

Time-resolved photoluminescence measurements were performed to monitor the decay of the PL band at about 580 nm corresponding to the Mn internal transition from the ^4^T_1 _excited state to the ^6^A_1 _ground state. For this purpose, the sample is excited with a light pulse of the third harmonic of an Nd-YAG laser (355 nm) with a pulse half-width of about 3.5 ns. The PL emission at various times after the excitation was recorded with an intensified charge-coupled device camera with a time resolution of 2 ns. The measurements were taken with the sample mounted inside a helium cryostat at 10 K.

## Results and discussion

**Figure 2:**
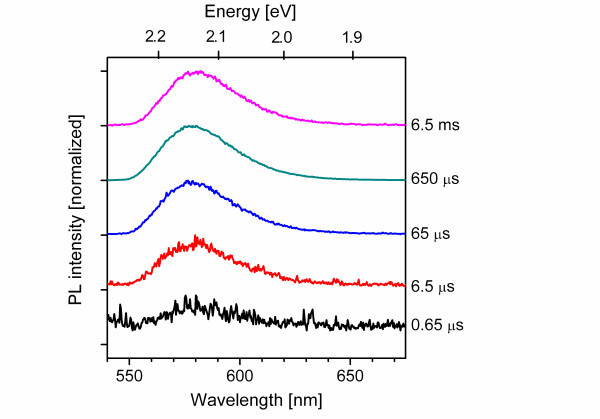
**Typical normalized PL spectra of a ZnS/Mn wire sample**. Recorded at different decay times after the laser excitation pulse; no energetic shift of the internal Mn luminescence is observed.

**Figure 3:**
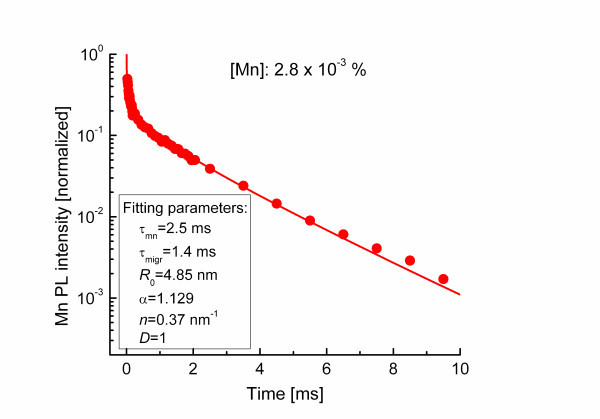
**The experimental data and the corresponding fitted curve from Mn PL transients**. ZnS/Mn wire samples with a Mn concentration of 2.8·10^-3 ^at.%.

The Mn internal transition of ZnS/Mn typically shows up as a single band in the yellow range of the visible spectrum. The intensity of the Mn yellow PL spectra measured at different times after the pulsed excitation were recorded, and their intensities are plotted in the decay curve against the time after arrival of the laser pulse on the sample. We are able to follow the decay of the Mn luminescence over more than four orders of magnitude in intensity in a decay time window ranging from 1 μs to 10 ms. Figure 2 shows a series of normalized PL spectra of the yellow Mn internal transition taken at different times after the laser excitation pulse. The time values given in the figure correspond to the center of an integration time window during the decay process, which was gradually extended with the decaying time. The integration time window was set to 1 μs for the early decay times below 20 μs. It has been extended to 10 μs for all measurements between 20 and 200 μs decay times to 100 μs for all measurements between 200 μs and 2 ms decay times and finally extended to 1,000 μs for all curves taken at later decay times. The broad luminescence band centered at 580 nm is characteristic for the internal luminescence of Mn^2+ ^incorporated on Zn sites. The symbols in Figure 3 show the decay of the Mn PL intensity of a ZnS/Mn wire sample which was implanted at 600°C. The Mn concentration of this sample is 2.8·10^-3 ^at.%, which corresponds to a large mean distance between Mn ions of about 11.4 nm, consequently, with a low resonant energy transfer effect between the Mn atoms. The transient is characterized by a rapid decrease shortly after the laser pulse followed by a slow, but rather, exponential tail at later times.

In the case of bulk ZnS/Mn samples with low Mn content, the transients of the internal Mn PL can be well described by the so-called Förster model [5] and its extension [10] based on dipole-dipole interaction between Mn ions and defect-related killer centers. At higher Mn concentration in bulk ZnS/Mn, the effect of the excitation transfer between Mn^2+ ^ions and killer centers on the transients of the internal Mn PL can be described by a modified Förster model for the 3D case [2].

For the 1D or 2D nanowires or nanoribbons [11], we developed a generalized formula for the Förster model. It was assumed that the dipole-dipole interaction is proportional to *R*^-6 ^where *R *is the distance between the Mn ion and the killer center. *R*_0 _is a critical length describing the strength of interaction and is defined as the distance between the Mn ion and the killer center where the dipole-dipole transfer rate from the excited Mn ion to the killer center equals the radiative decay rate of an isolated Mn ion. A value of *R*_0 _= 4.85 nm was determined by studying the Mn internal PL decay of ZnS/Mn spherical particles with diameters below 10 nm [6]. We obtain for the transient <*I*(*t*)/*I*_0_> of the Mn internal transition:(1)

where *D *is the dimensionality of the system, τ_Mn _is the decay time of an isolated Mn^2+ ^ion in ZnS, *n *= *N*/*R_g _*is the line density of killer centers. *R_g _*is the sample extension in one, two, or three dimensions, and α is a constant taking the values 1.129, 1.354, and √π for *D *= 1, 2, or 3, respectively. The parameter τ_migr _describes the effectiveness of the migration of the excitation within the Mn subsystem. For small Mn concentrations τ_migr _= τ_Mn_. The higher the Mn concentration *x*, the faster is the energy migration and the smaller is τ_migr_.

The transients of the wire-like samples in Figure 3 can be fitted perfectly by using our modified Förster model (Equation 1) with *D *= 1. The other parameters (see above) are set to *R*_0 _= 4.85 nm, τ_Mn _= 2.5 ms, *n *= 0.37 nm^-1^, and τ_migr _= 1.4 ms. Since τ_migr _is smaller than τ_Mn_, it confirms that energy migration takes place in the Mn subsystem, i.e., the Mn ions can no longer be considered as isolated. The value *D *= 1 shows that the energy transfer of the nanowires, which have a mean diameter of about 100 nm and a length of more than 10 μm behave like a 1D system due to the interplay of the lateral extension and mean distance between Mn centers. It confirms that the restricted dimensionality of the nanowire has a strong impact on the Mn PL decay behavior. The parameter *n *= 0.37 nm^-1 ^corresponds in 1D to a line density which, when accounting for the diameter of the nanowire of 100 nm, corresponds to a volume density *n*_killer _= 4.7·10^16 ^cm^-3^. This killer density in turn corresponds to a mean distance between killer centers of about 30 nm. As aforementioned, the mean Mn-Mn distance is 11.4 nm, thus, all the characteristic lengths, e.g., wire diameter and mean distances, are of the same order of magnitude. The system can be considered as quasi one-dimensional with a preferential energy transfer along the wire axis.

**Figure 4:**
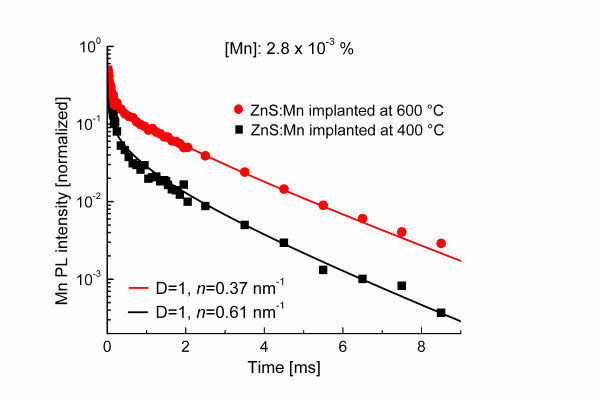
**The experimental data and the fitted curves from Mn PL transients**. Two ZnS/Mn wire samples with a Mn concentration 2.8·10^-3 ^at.% which were implanted at temperatures of 400°C and 600°C, respectively.

In Figure 4, the transients of two ZnS/Mn samples implanted at temperatures of 400°C and 600°C during the Mn implantation process (approach 1) with concentrations of 2.8·10^-3 ^at.% are shown. It can be seen that the Mn PL decay from the samples implanted at 400°C is faster than the decay of that at 600°C. This is in agreement with the expectation that with decreasing implantation temperature, the number of defects - thus killer centers - will increase. The curves can be fitted perfectly using the modified Förster model. By fitting with *D *= 1, *n *= 0.37 nm^-1 ^or 0.61 nm^-1^, and keeping all other parameters the same, one obtains volume killer densities of 7.8·10^16 ^cm^-3 ^and 4.7·10^16 ^cm^-3 ^for the samples implanted at 400°C and 600°C.

As we showed in an earlier paper [11] in detail, the effective dimensionality increases in case of higher Mn concentration although the wire diameter remains the same. We, therefore, checked the dependence of the modified Förster model on the killer density for wires with a substantially higher Mn concentration.

**Figure 5:**
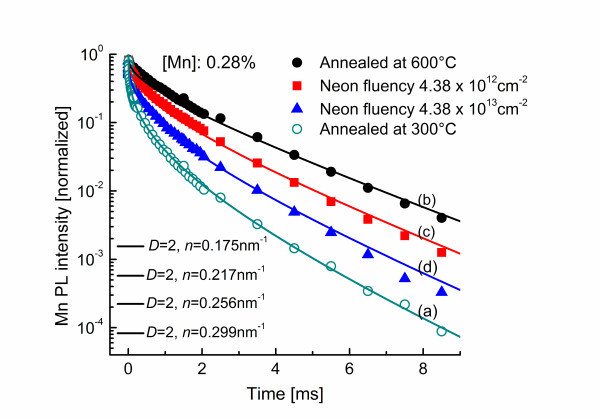
**The experimental data and the fitted curves from Mn PL transients**. ZnS/Mn wire samples with a Mn concentration 0.28 at.% which were implanted at temperatures of **(a) **300°C and **(b) **600°C as well as samples with additional neon irradiation by a total ion fluence of **(c) **4.38·10^12 ^cm^-2 ^and of **(d) **4.38·10^13 ^cm^-2^.

Figure 5 depicts the transients of various ZnS/Mn samples with a Mn concentration of 0.28 at.% but different killer center densities. The variation of the killer center densities is achieved by two different approaches. The curves (a) and (b) show Mn PL decay curves of ZnS/Mn nanowires annealed at 300°C and 600°C, respectively (approach 2). The curves (c) and (d) show the nanowires which were annealed at 600°C followed additional neon irradiation (approach 3) with a total ion fluence of 4.38·10^12 ^cm^-2 ^(curve c) and ion fluence of 4.38·10^13 ^cm^-2 ^(curve d).

Compared to annealing at 300°C, annealing at 600°C strongly reduces the number of defects in the ZnS/Mn nanowires. More defects in the sample means that it is easier for a Mn ion to find a killer center to transfer its excitation energy and the Mn PL intensity decays, therefore, faster. This trend is clearly shown in Figure 5. On the other hand, the neon implantation process after annealing at 600°C introduces additional lattice defects in the nanowires. The higher the neon fluence, the higher is the killer center density in the sample and the faster the Mn PL intensity decays, which is clearly shown in Figure 5.

The best fitting of all the four decay curves with the modified Förster model was obtained by using the effective dimensionality *D *= 2. The increasing of the effective dimensionality in the samples with higher Mn contents is as expected, since the average distance between Mn ions is now only about 2.5 nm. In a wire of a given diameter of 100 nm, the energy transfer is not only possibly parallel, but also perpendicular to the wire axis. It is, therefore, anticipated that the migration of the excitation energy inside the Mn subsystem is not a pure one-dimensional process anymore. It is worth mentioning that the value *D *= 2 should not be interpreted as that the energy transfer is only on a plane. It just corresponds to an effective dimension between 1D and 3D in the energy transfer process in the system.

The change of the killer center density is convincingly reflected by the transients of the Mn PL in Figure 5 and the fit parameters. The respective value of line densities of killer centers used by the fitting are *n *= 0.175 nm^-1 ^for the sample with the lowest number of defects (curve b), *n *= 0.217 nm^-1 ^(curve c), and *n *= 0.256 nm^-1 ^(curve d) for the neon implanted samples; *n *= 0.299 nm^-1 ^(curve a) for the sample annealed at 300°C, and this sample exhibits the highest line density as anticipated. Accounting for the geometry of the wire, the corresponding volume killer center densities are obtained as the following: 6.1·10^17 ^cm^-3 ^for the sample annealed at 600°C (b), 9.4·10^17 ^cm^-3 ^for the sample implanted with lower neon fluences (c), 1.3·10^18 ^cm^-3 ^for the sample implanted with higher neon fluences (d) and 1.8·10^18 ^cm^-3 ^for the sample annealed at 300°C (a), respectively.

## Conclusions

We have demonstrated that the PL transient of the Mn internal transition from the ^4^T_1 _excited state to the ^6^A_1 _ground state becomes faster when additional defects acting as killer centers are introduced in the ZnS/Mn nanowire samples. The number of killer centers can be increased either by decreasing the sample temperature in the Mn implantation process, by decreasing the post-annealing temperature, or by an additional implantation process using Neon ions. The variation of the PL transients with killer center density can be quantitatively described using the modified Förster model for low dimensionality introduced previously. An almost perfect fit for various killer center densities was possible for wires with rather low Mn content (*D *= 1) and higher Mn concentrations (*D *= 2). Furthermore, we were able to confirm that the effective dimensionality is determined by the interplay of the average distance between the Mn ions, the killer center densities, and the spatial extensions of the nanowires.

## Competing interests

The authors declare that they have no competing interests.

## Authors' contributions

UK carried out the Time-resolved PL measurements, data analysis and interpretation. LC participated in the optical measurements, data interpretation and drafted the manuscript. SG carried out the sample growth, ion implantation work, TEM study and revised the manuscript. CR and WH conceived the study, participated in the data interpretation and revised the manuscript critically.
